# Isolated Posterior Malleolar Fracture: A Case Report of a Rare Presentation With Narrative Review of Literature

**DOI:** 10.7759/cureus.21658

**Published:** 2022-01-27

**Authors:** Lavindra Tomar, Gaurav Govil, Pawan Dhawan

**Affiliations:** 1 Department of Orthopaedics, Max Super Specialty Hospital, Patparganj, Delhi, IND

**Keywords:** syndesmotic screw fixation, malleolar fracture, missed, isolated, posterior malleolar fracture, ankle fracture

## Abstract

The isolated posterior malleolar fracture is a rare case. An innocuous injury may have associated ligamentous disruption. The fracture classification and treatment protocol are not well-defined. A missed injury results in poor functional outcomes.

A 28-year-old male sustained a twisting injury to his left ankle. The radiograph revealed an isolated posterior malleolar fracture. A computerized tomographic examination suggested talus lateralization and deltoid ligament injury. Surgical fixation with a syndesmotic screw was done. Post-operative delayed weight-bearing was allowed. At a one-year follow-up, there was painless weight-bearing and independent mobilization.

The posterior malleolus significantly contributes to ankle stability. The estimation of fragment size may be an erroneous guiding factor for surgical fixation. Recent literature suggests that syndesmotic stability, residual talus subluxation, joint congruence, and fibular notch involvement are more significant factors for risk assessment and to guide the management of posterior malleolar fracture.

The isolated posterior malleolar fracture presents rarely. They should be evaluated by tomographic evaluation and an unstable injury should be surgically managed.

## Introduction

An isolated posterior malleolar fracture (PMF) presents rarely, accounting for 0.5-1% of all ankle fractures [1,2]. The earliest description has been given as a “parachute jumper's fracture” [3].

Plain ankle radiographs may be insufficient to identify an isolated injury of PMF. Studies have shown a high probability of missed fractures [4]. The computerized tomography (CT) evaluation with 3D reconstruction will allow a valid assessment of suspect posterior malleolar ankle injuries to further classify and treat them effectively [2,5,6].

The posterior malleolus contributes significantly to the ankle stability as it contributes to tibiotalar load transfer and any disruption may induce a talus subluxation posteriorly [7]. This needs to be accurately assessed for an effective functional outcome. A review of biomechanical studies stressed the significant contribution of the tibiofibular ligament as a predominant posterior ankle stabilizer [8]. An inadequate fracture stabilization, any neglected or missed injury, ankle instability may contribute to poor functional outcomes with the propensity for an early onset of secondary arthritis of the ankle joint [7-9].

The article aimed to highlight the innocuous nature of an isolated PMF liable for poor outcomes with conservative management. We also review the literature for an isolated PMF and narrate the current treatment protocol and recommendations for its management.

## Case presentation

**Figure 1:**
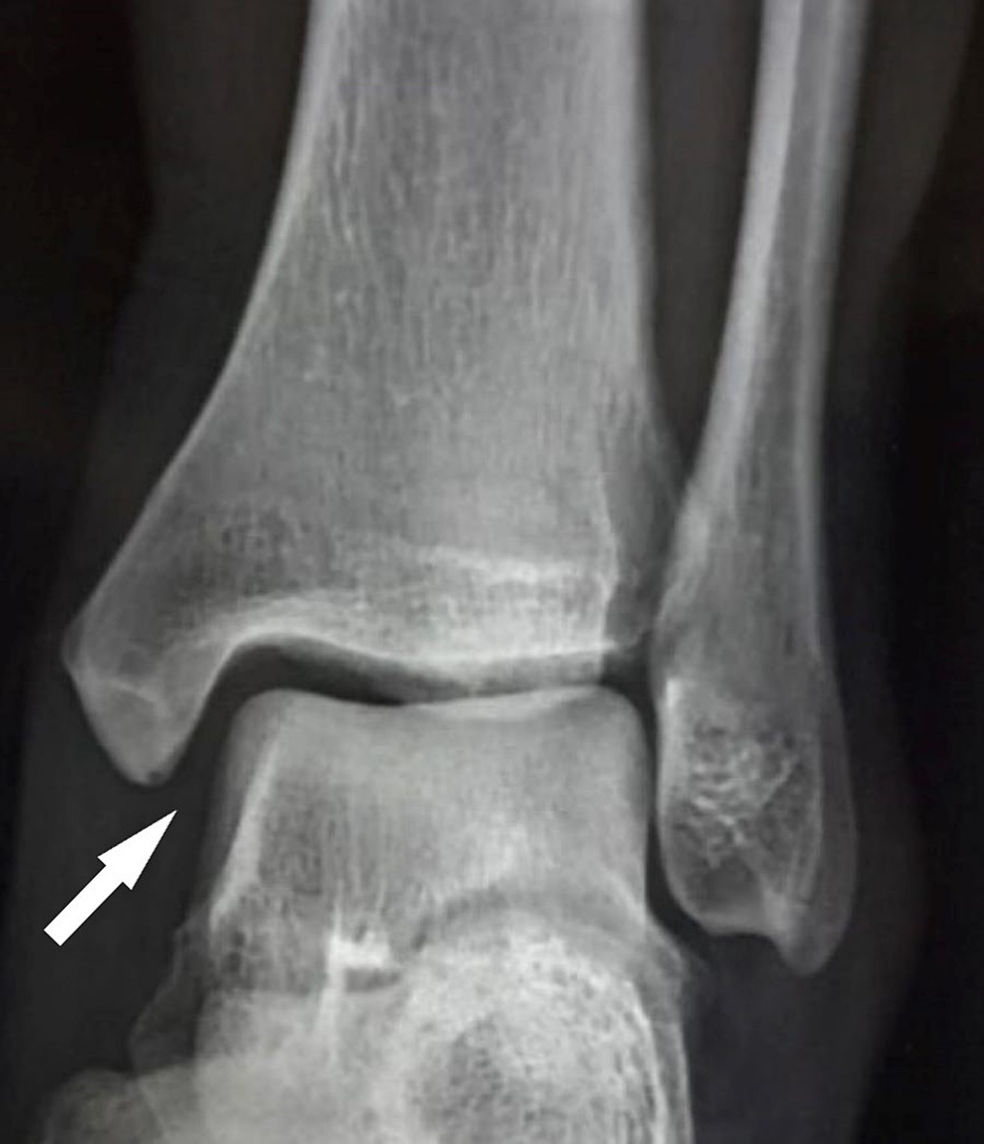
Antero-posterior view radiograph of the left ankle shows an increased medial clear space (white arrow) of the ankle joint.

**Figure 2:**
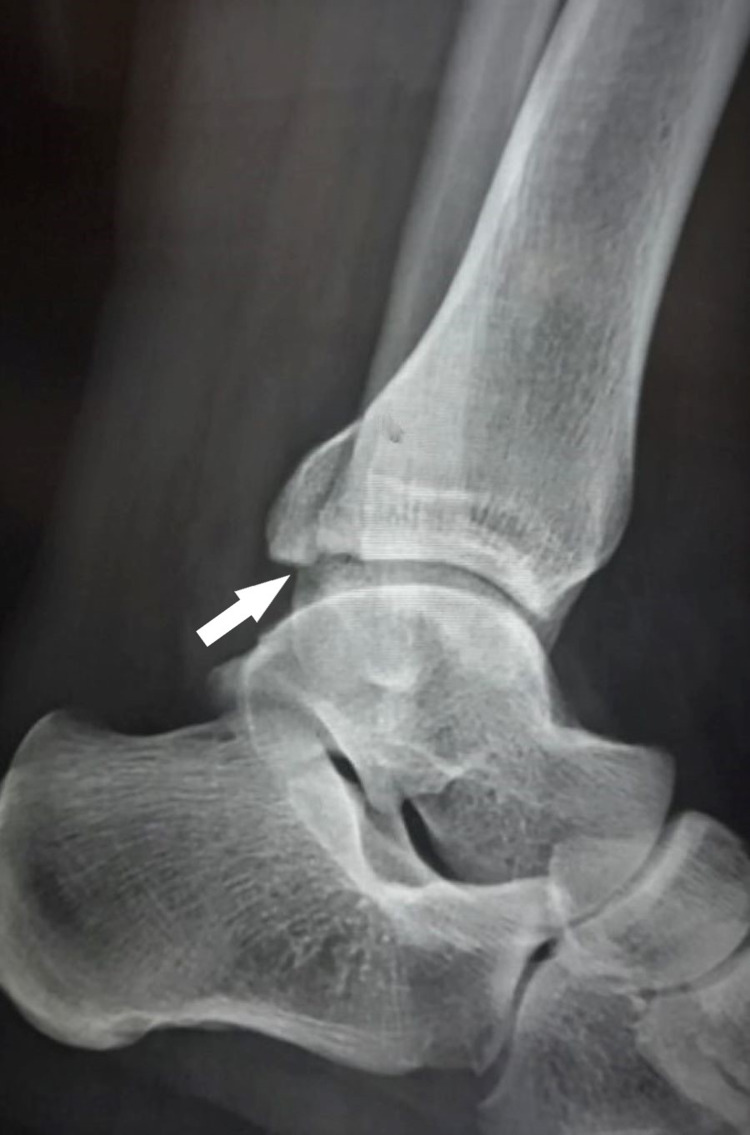
Lateral view radiograph of the left ankle shows posterior malleolar fracture (white arrow) with less than 25% involvement of tibiotalar articulation.

A 28-year-old student sustained an injury to the left ankle region due to twisting while walking on an uneven surface. Weight-bearing was painful but he limped with a swollen ankle and painful movements to the emergency department for evaluation. Tenderness was localized along the medial malleolar region. There was no bruising, contusion, or skin blistering. Ankle range of movements was painful and restricted. The plain ankle radiograph revealed an isolated PMF with the opening of the medial joint space with talus tilting laterally suggesting deltoid ligament disruption medially (Figures 1, 2).

**Figure 3:**
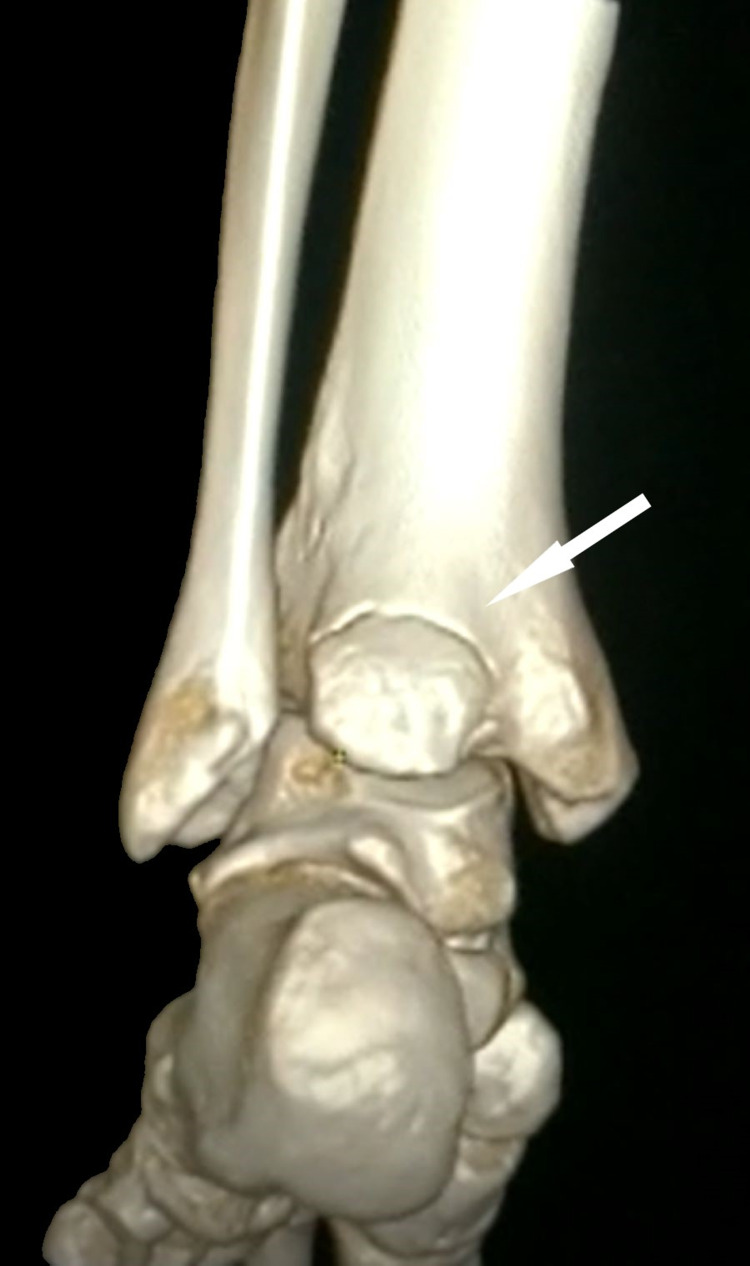
3D reconstruction computerized tomographic scan of the left ankle delineates the posterior malleolar fracture fragment (white arrow).

**Figure 4:**
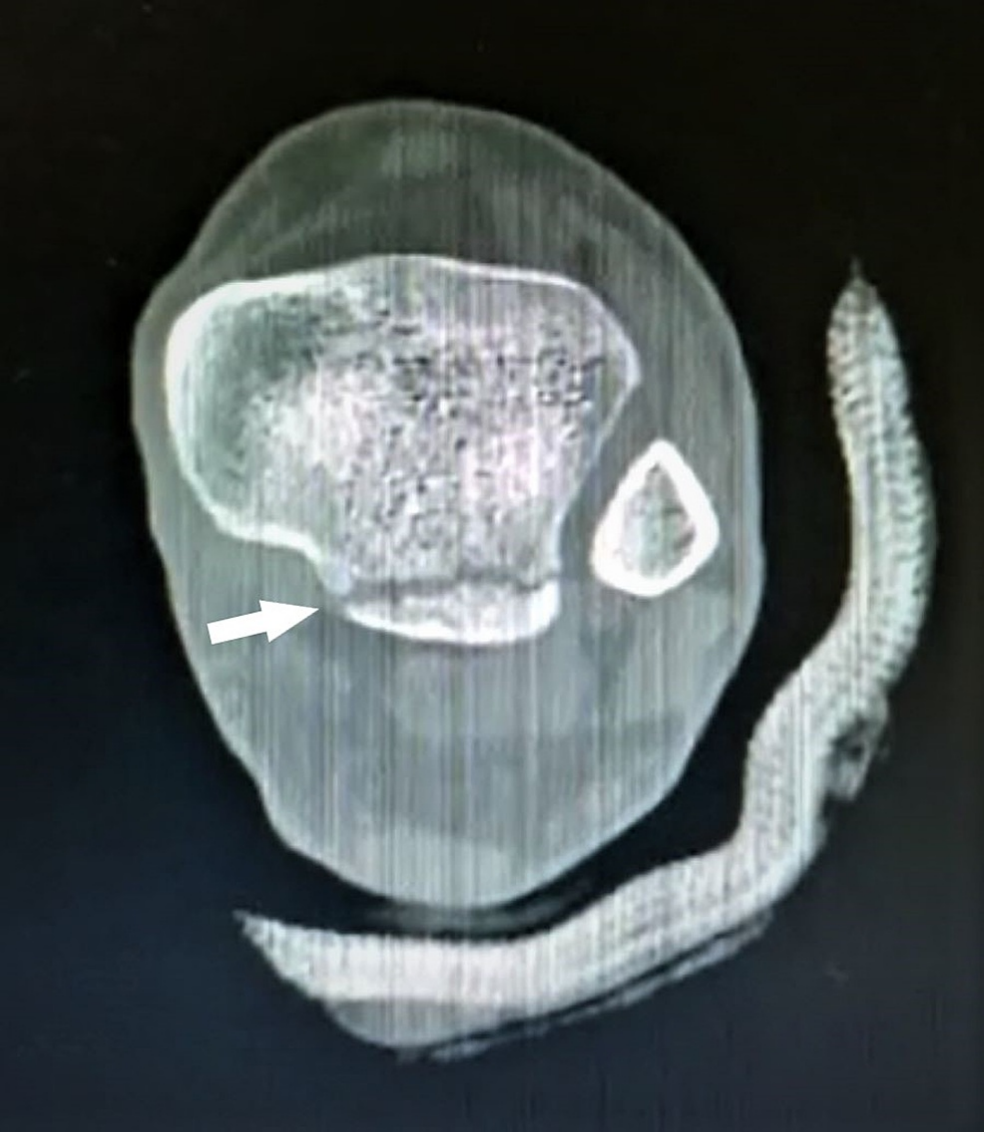
Computerized tomography scan of the left ankle shows less than 25% involvement (white arrow) of the articular surface.

The non-contrast CT with 3D reconstruction was done to further assess the injury and it confirmed the injury to be an isolated PMF. The fragment was less than 25% of the articular surface with talus lateralization indicating the syndesmotic injury (Figures 3, 4).

**Figure 5:**
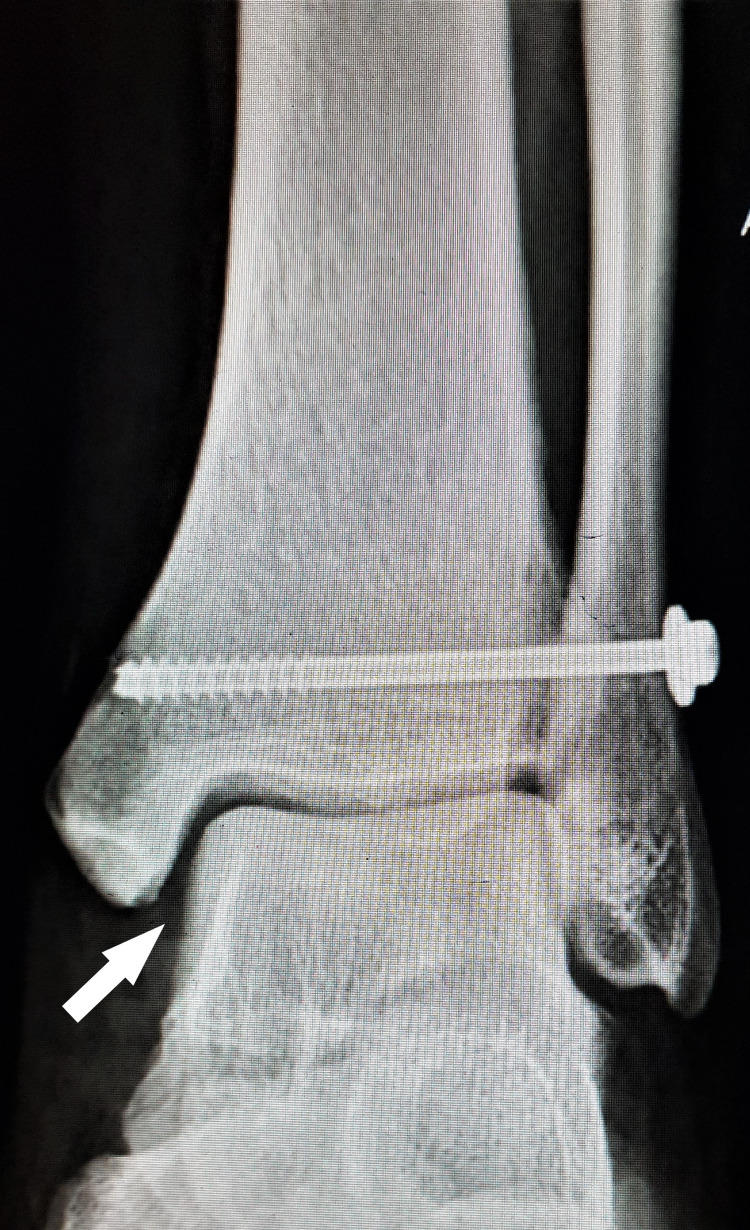
Postoperative antero-posterior view radiograph of the left ankle at three months, shows a reduced ankle joint with normal medial clear space (white arrow).

**Figure 6:**
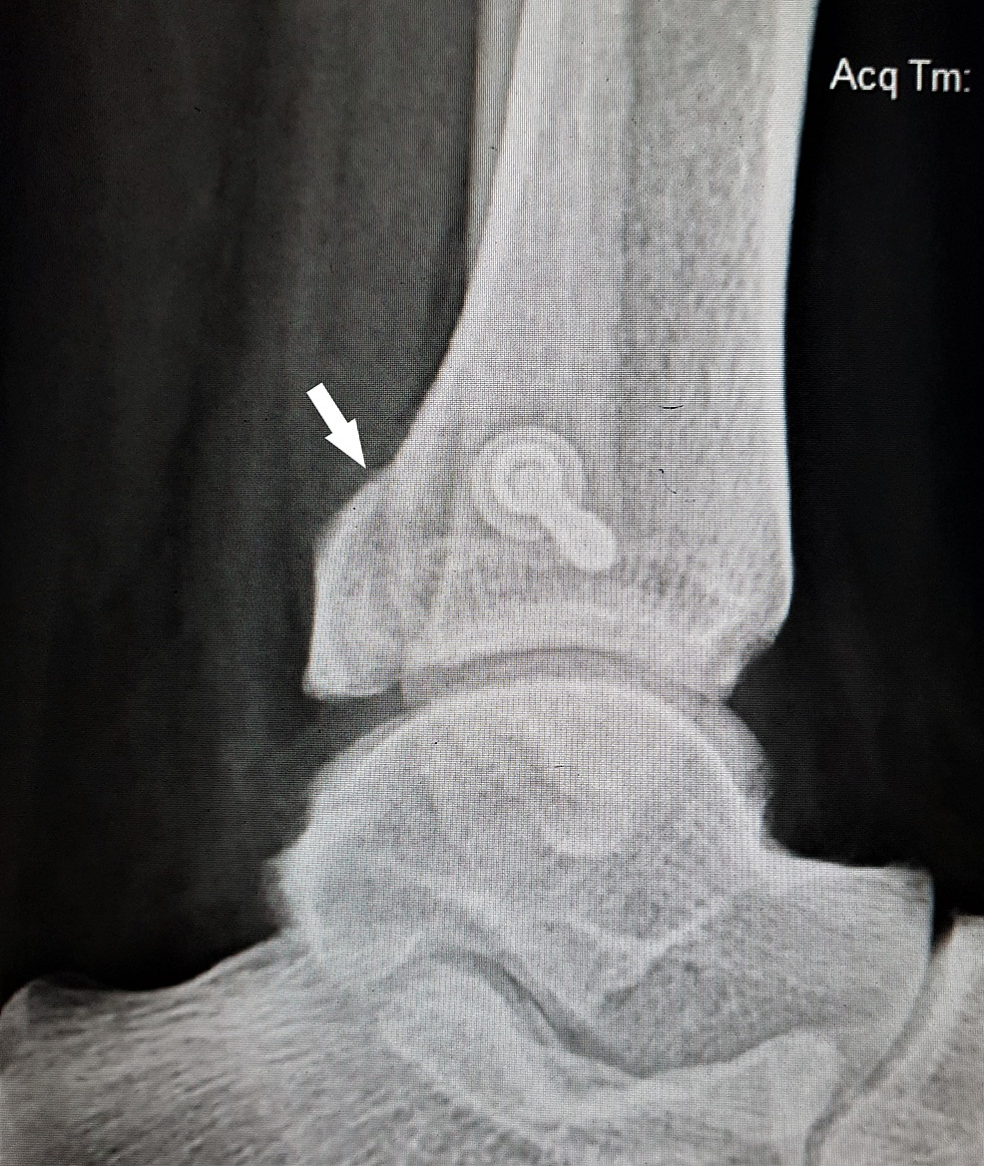
Postoperative lateral view radiograph of the left ankle at three months, shows united posterior malleolar fracture (white arrow).

The surgical management was done to restore the ankle joint congruity and its stability. The surgical management with a syndesmotic 4.5 mm cannulated cancellous screw fixation under image intensifier guidance was done. Post-operative immobilization in a below-knee cast for six weeks with non-weight bearing walker support walking was allowed. After six weeks, ankle mobilization and partial weight-bearing for another one month with an elbow crutch support was advised. After ten weeks, full weight-bearing and un-assisted walking were allowed (Figures 5, 6).

At one-year follow-up, the ankle function was painless and the functional outcome has been graded as excellent.

## Discussion

The isolated PMF presents rarely. The fracture has a concomitant ligamentous injury which may be under-reported in literature [4]. They are innocuous-looking injuries presenting as simple ankle sprains with grave implications and poor outcomes [10]. The injuries are often missed, detected late, or neglected [1,4,10,11]. Even Ottawa ankle rules lack a description of specific tender points for suspect PMF [12].

The incidence of an isolated fracture has been reported from 0.5-4% of all the ankle fractures [10,11,13]. The males are predominantly affected [10,11]. An isolated bony injury has commonly associated soft tissue ligamentous injury [10,11,13].

The pathophysiological mechanism involves an axial loading on a fixed plantarflexed ankle or a rotational strain on the syndesmotic ligament [1,4,13]. The literature lacked a classification system for an isolated PMF based on plain radiology [6]. The Lauge-Hansen classification for ankle fractures does not include an isolated PMF [1,14]. Classification based on CT has been used to identify and treat PMF in recent literature [5,15].

The imaging of choice for an isolated PMF, therefore, has been a 3D reconstruction CT scan [8,9,11,14]. The stress radiographs may be considered for the evaluation of a syndesmotic injury. However, their use for the evaluation of PMF injury has been inconclusive and debatable [5,11]. Ligament injury and osteochondral lesions evaluation warrants magnetic resonance imaging (MRI) [1,5,10]. The goal has been to identify the extent and severity of the injury.

A study with a systematic review identified 45 cases of isolated PMF between 1996 and 2014 managed conservatively with good functional outcomes [8]. Another systematic review identified 44 cases of isolated PMF between 1978 and 2014 based majorly on two studies with little contribution to isolated PMF from other studies [9]. An isolated injury management protocol was not discussed due to heterogenicity in the studies. The review study till 2016 literature similarly identified a total of 75 cases of isolated PMF injuries. Eighty-five percent were managed by conservative management in cast immobilization with a normal recovery [11].

Normally, the decision-making for a conservative or surgical management approach for PMF has been based largely on the size of the posterior malleolar fragment [11,16]. The common dictum in practice has been to consider conservative management unless the fracture involves 25% or more of the articular surface or has a more than 2 mm step-off as clear indications for reduction and fixation of posterior fragment [1,2,4,9,10,14]. However, systematic reviews have concluded that the fragment size of PMF does not correlate to the outcomes obtained [8,9].

Recent literature considers the ankle syndesmotic stability, residual talar subluxation, joint congruence, and fibular notch involvement as more significant guiding factors for deciding the management strategy and need for surgical fixation of PMF [2,5]. If the talus is stable or the medial and lateral malleoli are not affected, cast immobilization can be given for an isolated PMF [10]. Any instability around the ankle needs consideration for surgical management.

The techniques used for fixation are varied. The CT-based classification guides regarding the fixation techniques and approaches for surgical fixation [16]. A temporary spanning external fixator may be required to align the ankle joint and allow for soft tissue healing preceding the final surgical fixation [16]. The syndesmotic injuries are surgically treated with syndesmosis screw fixation [17]. An anchor button along with PMF fixation has also been used depending on the size of the PMF fragment [16]. The anteroposterior lag screw fixation has been used commonly with indirect reduction of fracture along with cast immobilization [14,18]. The posteroanterior lag screw fixation with direct reduction has been advised with cast immobilization for better control and congruent fixation of the fragment [1]. The posterolateral approach has been identified as the gold standard approach. Through this approach in the prone position, an anti-glide or buttress plate fixation technique for PMF has been advocated [2,10,14]. The posteromedial approach alone or in combination with the posterolateral approach can also be used for more complex or medial-based injury patterns [16]. The syndesmotic disruption requires fixation for an appropriate tibiofibular alignment whilst ligaments heal either with a single or double syndesmotic screw [19].

A cadaveric study provides comprehensive biomechanical analysis for the three fixation techniques namely posteroanterior lag screw fixation, anteroposterior lag screw fixation, and plate fixation for PMF, and concludes with superiority of plate fixation with less cut-out and loss of fixation evident with other two techniques [7].

The outcomes reported in the literature are variable for an isolated PMF with both conservative management and surgical fixation. The consensus for surgical management has gained preference for an optimal functional outcome [14].

## Conclusions

An ankle sprain on initial evaluation with a plain radiograph, may not match with clinical presentation. An innocuous ankle injury may have an occult bony or ligament injury which needs to be further assessed with stress radiographs, CT or MRI imaging. Any neglected or missed ankle injury may likely result in a poor functional outcome.

An isolated PMF needs to be identified early. A timely diagnosis and surgical management for an unstable isolated PMF will give a favorable functional outcome.
